# Exploring decision-making strategies in the Iowa gambling task and rat gambling task

**DOI:** 10.3389/fnbeh.2022.964348

**Published:** 2022-11-03

**Authors:** Cathrine Hultman, Nikita Tjernström, Sofia Vadlin, Mattias Rehn, Kent W. Nilsson, Erika Roman, Cecilia Åslund

**Affiliations:** ^1^Centre for Clinical Research, Västmanland Hospital Västerås, Region Västmanland, Uppsala University, Västerås, Sweden; ^2^Department of Public Health and Caring Sciences, Uppsala University, Uppsala, Sweden; ^3^Department of Pharmaceutical Biosciences, Uppsala University, Uppsala, Sweden; ^4^School of Health, Care and Social Welfare, Mälardalen University, Västerås, Sweden; ^5^Department of Anatomy, Physiology and Biochemistry, Swedish University of Agricultural Sciences, Uppsala, Sweden

**Keywords:** decision-making, Iowa gambling task, rat gambling task, uncertainty, risk

## Abstract

Decision-making requires that individuals perceive the probabilities and risks associated with different options. Experimental human and animal laboratory testing provide complimentary insights on the psychobiological underpinnings of decision-making. The Iowa gambling task (IGT) is a widely used instrument that assesses decision-making under uncertainty and risk. In the task participants are faced with a choice conflict between cards with varying monetary reinforcer/loss contingencies. The rat gambling task (rGT) is a pre-clinical version using palatable reinforcers as wins and timeouts mimicking losses. However, interspecies studies elaborating on human and rat behavior in these tasks are lacking. This study explores decision-making strategies among young adults (*N* = 270) performing a computerized version of the IGT, and adult outbred male Lister Hooded rats (*N* = 72) performing the rGT. Both group and individual data were explored by normative scoring approaches and subgroup formations based on individual choices were investigated. Overall results showed that most humans and rats learned to favor the advantageous choices, but to a widely different extent. Human performance was characterized by both exploration and learning as the task progressed, while rats showed relatively consistent pronounced preferences for the advantageous choices throughout the task. Nevertheless, humans and rats showed similar variability in individual choice preferences during end performance. Procedural differences impacting on the performance in both tasks and their potential to study different aspects of decision-making are discussed. This is a first attempt to increase the understanding of similarities and differences regarding decision-making processes in the IGT and rGT from an explorative perspective.

## Introduction

Decision-making is a process involving a choice between two or more different alternatives, which requires that individuals perceive the probabilities and risks associated with each option and estimate the consequences of each option in the short, medium, and long term (Ernst and Paulus, 2005; Mata et al., 2011; Buelow, 2020). Decision-making impairments can be defined as a tendency toward risky or unwise choices and play an important role in substance use disorders and behavioral addictions (Bickel et al., 2018; Koffarnus and Kaplan, 2018), as well as other psychiatric conditions or neuropsychiatric disabilities (Buelow and Suhr, 2009), such as schizophrenia (Kim et al., 2016), obsessive compulsive disorder (Zhang et al., 2015), attention-deficit hyperactivity disorder (Toplak et al., 2005), and affective disorders (Miu et al., 2008; Must et al., 2013; de Siqueira et al., 2018). One way of conceptualizing decision-making is through experimental studies using laboratory behavioral tests on both humans and animals. Studies have shown that decision-making in animals share similar preferences and biases that are seen in human choice behavior, e.g., escalating commitment and loss chasing (Winstanley and Clark, 2015). These similarities enable the use of laboratory animals to understand the neurobiological underpinnings of decision-making. Several rodent tasks have been developed to enable studies of different aspects of decision-making (de Visser et al., 2011; Izquierdo and Belcher, 2011; Winstanley and Floresco, 2016; Izquierdo et al., 2019). Such tasks aspire to simulate human decision-making processes. However, studies examining interspecies data on human and animal behavior in these tasks are scarce, but important from a translational perspective.

The Iowa gambling task (IGT) (Bechara et al., 1994) is a widely used clinical and experimental instrument for the assessment of decision-making under uncertainty and risk. The task requires individuals to perceive risk probabilities through feedback of monetary reinforcers and punishment to achieve the optimal decision-making strategy. The task was originally designed to measure decision-making impairments among patients with damage to the ventromedial prefrontal cortex (VMPFC) (Bechara et al., 1994, 1997, 1999, 2000a) and has since been used to evaluate decision-making performance among a wide range of both clinical and non-clinical human samples (Buelow and Suhr, 2009). It is also a valid measure of decision-making in gambling disorder (Brevers et al., 2013; Linnet, 2013). In the IGT, participants are presented with four decks with each card containing monetary reinforcers and occasional penalties. Unknown to the participants the cards differ in their monetary gain/loss schedule and long-term gain probability. Two of the decks (A and B) contain large immediate gains, but these are offset by larger occasional penalties. Continuous selections from these decks results in an overall monetary loss, making them disadvantageous with regards to their long-term outcome. The other two decks (C and D) are advantageous and provide small gains and small penalties, resulting in a long-term monetary profit. Hence, the optimal decision-making strategy, resulting in the participant maximizing the total gain, is achieved through learned preferences for the long-term profit. Impaired decision-making is characterized by preferences for the riskier options and larger immediate rewards, suggesting an insensitivity to future consequences or a hypersensitivity to reward (Bechara et al., 2000a,^2002^; Bechara and Damasio, 2002). The standard approach to assess performance on the IGT is by subtracting disadvantageous (deck A + B) from advantageous (deck C + D) choices across 100 trials or per 20-trial blocks, resulting in individual net-scores indicating a tendency toward advantageous or disadvantageous choices (Bechara et al., 2000b). Research also suggests that some individuals in the IGT may apply a decision-making strategy where they seek to minimize the frequency of losses, rather than maximizing the long-term gains of the different choices (Caroselli et al., 2006; Lin et al., 2007; Chiu et al., 2008; Steingroever et al., 2013; Barnhart and Buelow, 2021). This is indicated by net-scores obtained from the number of choices from the low-loss frequency decks (B + D) minus the high-loss frequency decks (A + C).

During the first part of IGT, participants have insufficient knowledge about the risks and benefits associated with each option. The participants acquire information on the relative risks and benefits based on the reinforcer and punishment feedback obtained as the task progresses (Brand et al., 2007). Early research put forth a dual process theory to explain how decisions are made in the IGT, suggesting that decisions are guided by emotions and “gut feelings” during the early trials, i.e., decisions under ambiguity, and as the task progresses, more calculated and conscious decisions occur, i.e., decisions under risk (Maia and McClelland, 2004; Dunn et al., 2006; Brand et al., 2007; Guillaume et al., 2009). However, on an individual level, the gradual transition from implicit to more explicit processes in decision-making occurs at different time points in the task (Brand et al., 2007). Additionally, these systems do not operate independently, but interact to form decisions through connections between amygdala, striatum, VMPFC, and dorsolateral prefrontal cortex (DLPFC) (Bechara et al., 1999; Bechara and Damasio, 2005; Weller et al., 2007; Lin et al., 2008; Wood and Bechara, 2014). Hence, performance on the IGT is complex and relies on the interaction of both emotional feedback processing as well as more deliberate cognitive resources, although the extent to which these processes operate and interact to guide decisions varies to a great extent between individuals (Wood and Bechara, 2014; Buelow and Blaine, 2015). It is well known that healthy human individuals show considerable variability in their performance on the IGT indicated by widely diverging net-scores, tendencies to explore the different options, and varying learning rates (Steingroever et al., 2013; Bull et al., 2015; Barnhart and Buelow, 2021). The individual differences in IGT performance observed among non-clinical human samples may be attributed to several factors such as educational levels (Davis et al., 2008), motivation (Giustiniani et al., 2015), negative affect (Suhr and Tsanadis, 2007), as well as mood and personality characteristics (Buelow and Suhr, 2013).

The rat gambling task (rGT) is loosely based on the IGT and uses sucrose pellets as reinforcers and timeout periods as punishment, mimicking monetary losses in the IGT (Zeeb et al., 2009; de Visser et al., 2011). In contrast to the IGT, the time-outs in the rGT does not detract earnings, but rather the opportunity to earn pellets during a certain amount of time. Other rodent gambling tasks have used aversive tasting quinine pellets to signal a loss (de Visser et al., 2011). However, using this approach the rats never risk finishing the task with a disadvantageous outcome. The optimal strategy in IGT requires individuals to maximize their overall profit across the task. Similarly, in the rGT used herein, individuals have a limited amount of time to maximize the number of pellets and must learn to avoid the options associated with larger immediate number of reinforcers and longer punishing time-outs. Hence, both tasks assess strategies of overall maximization.

The rGT was originally developed to specifically assess gambling-like decision-making processes. It was designed to be suitable for experiments testing the effects of different manipulations, including pharmacological, on decision-making. In the rGT, rats perform repeated daily testing in order to reach stability in choice preferences (Zeeb et al., 2009). The outline of the test is similar to the IGT, with four choices associated with different gain/loss contingencies. However, options in the rGT differ from those in the IGT both regarding frequency and magnitude of gains and losses. In the IGT there are clearly two advantageous and two disadvantageous options with regards to their long-term net gain, while the choices in the rGT are harder to classify in that way. One choice is clearly the most advantageous, and the remaining three options are increasingly disadvantageous. We and others have shown that the majority of rats learn and maintain a stable choice on the most advantageous option associated with smaller immediate gains but greater overall net gain, in favor of the disadvantageous options associated with larger immediate gains but greater overall net losses (Zeeb et al., 2009; Zeeb and Winstanley, 2011; Barrus et al., 2015; Barrus and Winstanley, 2016; Tjernström et al., 2022). Decisions are based on reinforcer/punishment feedback gained during the task and studies have established that similar brain regions, as observed in humans performing the IGT (such as DLPFC and VMPFC/orbitofrontal cortex), are activated during the course of the rGT (Zeeb et al., 2015). In addition, individuals with different gambling strategies in the rGT were found to differ in connectivity in regions associated with brain reward networks (Tjernström et al., 2022).

Studies investigating inter-individual variability in decision-making using various versions of rodent gambling tasks found subpopulations of decision-makers (Rivalan et al., 2009; Pittaras et al., 2016; Cabeza et al., 2020; Tjernström et al., 2022), suggesting similar variability as observed in healthy humans (Steingroever et al., 2013). Similar to the standard scoring approaches commonly used in the IGT (Bechara et al., 2000b), many rGT studies tend to group the options into advantageous and disadvantageous choices, and the main focus has been on factors and treatments that attenuate the disadvantageous options in favor of the more advantageous choices (Rivalan et al., 2011; Zeeb and Winstanley, 2011; Adams et al., 2017a,b). Nevertheless, these scoring approaches fail to consider individual choice behavior and potential variability in the decision-making strategies within groups of healthy individuals. Therefore, studies on individual differences in the rGT are warranted.

Studies assessing and comparing interspecies data on the IGT, and rodent analogs are scarce. Cabeza et al. (2020) statistically compared human performance on the IGT and mouse performance on the IGT-adapted mouse gambling task (mGT), using the same analytical approaches. As indicated by previous research, similar patterns of choice strategies were found among humans and mice, where both species progressively developed a preference for the long-term advantageous choices, although mice showed a faster learning rate than humans (Cabeza et al., 2020). However, there are currently no studies comparing data from both human and rat performance on the IGT and the rGT in the same study, using the same scoring approaches. The rGT is often referred to as a rat analog to the IGT even though there are considerable procedural differences between the human and rat tasks, and it is not clear to what extent the different choices on the rGT corresponds to the choices of the IGT. Reporting the results from both human and rat experiments and unraveling different aspects of decision-making involved during these tasks may provide valuable insights for both clinical and pre-clinical researchers using these tasks.

Therefore, the aim of this study was to explore individual decision-making strategies in experimental gambling settings among humans and rats. Initially, the choices of the IGT and rGT were investigated using the grouping method that is similar for both tasks, where scores are calculated by subtracting disadvantageous from advantageous choices (Bechara et al., 2000b; Zeeb and Winstanley, 2011). Furthermore, the individual choices were explored by comparing subgroup formations for identification of strategic and non-strategic decision-making individuals.

## Materials and methods

### Procedures of the Iowa gambling task

#### Human participants

Human participants of this study were recruited from a large cohort study [Survey of Adolescent Life in Västmanland, SALVe Cohort; the 2015 wave 2 (*n* = 1,644)] of young adults, born in 1997 and 1999. Data were collected during an experimental session at Västmanland County Hospital, Västerås, Sweden. Eligible participants, based on criteria required for the experimental session, were consecutively included until the final sample was reached [for a detailed report on the inclusion procedure, see Hultman et al. (2022)].

In total, 270 volunteers (140 women, 130 men) aging between 18 and 22 years were invited to an experimental gambling session. Data were collected during 2017–2019. Upon direct questioning, none reported any current or previous history of gambling disorder diagnosis. Prior to the experimental session the participants also completed the Problem Gambling Severity Index (PGSI) (Ferris and Wynne, 2001). The scores were summarized in four categories of different levels of problem gambling: non-problem gambler = 0, low-risk gambler = 1–2, moderate-risk gambler = 3–7, problem gambler ≥8 (Public Health Agency of Sweden, 2010). According to self-reports, 35 participants fell in the categories from low-risk gambler to problem gambler, with 20 participants considered as low risk (2 females, 18 males), 14 as moderate risk (3 females, 11 males) and one male considered a problem gambler (Public Health Agency of Sweden, 2010). Independent sample *t*-tests were performed to compare the sub-sample of the current study (*N* = 270) and the cohort (*N* = 1,215), in terms of self-reported symptoms on the Adult ADHD Self-Report Scale (ASRS) (Kessler et al., 2005), Depression Self-Rating Scale (DSRS) (Svanborg and Ekselius, 2003), and the Adult Anxiety Scale-15 (AAS-15) (Spence, 2017). No significant differences were found in terms of self-reported symptoms of depression (*p* = 0.961) or ADHD (*p* = 0.543), while the current sub-sample had significantly lower levels of self-reported symptoms of anxiety compared to the cohort (*p* = 0.015).

Detailed information on the procedure was given by the examiner and informed consent was obtained from all participants upon arrival to the experimental session. The study was approved by the Ethical Review Board of Uppsala (dnr 2016/569), with an extended approval (dnr 2019-01368).

#### Experimental procedure

Human participants performed several tasks during the same experimental session. The sessions included: a battery of questionnaires (on gambling, gaming, personality traits, sleep habits, sensory processing sensitivity, and positive/negative affect), four emotion cognition tasks, two interviews on substance- and behavioral addictions, and three different gambling tasks. Participants were reimbursed a gift card with a fixed amount (1,000 SEK ≈ 100 €) for participation. The participants were also informed that they would receive additional gratification depending on their performance in the gambling tasks. The maximum additional amount was 200 SEK/task (10 SEK ≈ 1 €). The IGT considered in the current study was administered as the second out of three gambling tasks during the latter part of the session.

#### Iowa gambling task

A computerized version of the original IGT by Bechara et al. (1994) was administered displaying four virtual disadvantageous (A and B) and advantageous (C and D) card decks, from which the participants repeatedly chose one card at a time in order to maximize their profit as much as possible across 100 trials. The original task was modified in terms of visual and auditory stimuli to resemble a casino environment. Similar to the published instructions (Bechara et al., 1999), the only hint provided about the task was that some decks were better, and some were worse. These instructions are known to improve performance on the task (Balodis et al., 2006; Fernie and Tunney, 2006). All participants started with a credit of 2,000 SEK. Two bars/lines showing the continuously accumulated amount of earnings and losses were displayed at the top of the computer screen, and the total earnings displayed to the right. Participants used the computer mouse to select a deck, revealing a card with two values: gain and loss. The sum on each card resulted in either a positive amount added to the total earnings, or a negative amount subtracted from the total earnings. A positive amount was followed by a winning sound, and conversely, a negative amount was followed by a losing sound. Participants could switch between each deck as many times as they pleased. Continuous selections from decks A or B over 10 trials resulted in a net loss of 250 SEK, while 10 selections from decks C or D resulted in a net gain of 250 SEK. The win-loss-contingencies during the first 10 trials (and repeated throughout the task) are presented in Table 1. The total virtual net profits at the end of the task were converted to a real gift card gratification according to the following: net profits <100 SEK resulted in 0 gratification, net profits between 100 and 1,000 SEK resulted in 100 SEK gratification, and net profits >1,000 SEK resulted in 200 SEK gratification.

**TABLE 1:** The win-loss-contingencies (in SEK) during the first 10 trials and repeated throughout the IGT.

Deck	A		B		C		D	
				
Trial	Gain	Loss	Gain	Loss	Gain	Loss	Gain	Loss
1	100	0	100	0	50	0	50	0
2	100	0	100	0	50	0	50	0
3	100	−150	100	0	50	−50	50	0
4	100	0	100	0	50	0	50	0
5	100	−300	100	0	50	−50	50	0
6	100	0	100	0	50	0	50	0
7	100	−200	100	0	50	−50	50	0
8	100	0	100	0	50	0	50	0
9	100	−250	100	−1,250	50	−50	50	0
10	100	−350	100	0	50	−50	50	−250
Net gain	−250		−250		+250		+250	
Frequency wins/losses	10 wins 5 losses		10 wins 1 loss		10 wins 5 losses		10 wins 1 loss	

### Procedures of the rat gambling task

#### Animals and housing

The rat data used herein are combined based on two previous studies (Tjernström et al., 2022; Tjernström and Roman, 2022; manuscript resubmitted for review). Outbred male Lister Hooded (HsdOla:LH, Envigo, Horst, Netherlands; *N* = 72) rats were delivered at 5–6 weeks of age. The animals were pair-housed in transparent cages type IV (59 × 38 × 20 cm) with raised lids containing wood chip bedding. For enrichment purposes each cage had paper sheets (40 × 60 cm, Cellstoff, Papyrus) and a wood tunnel. The cages were kept in an animal room on a reversed light/dark cycle (lights off at 6:00 am) with a masking background noise. The animal room was kept in constant temperature (22 ± 1°C) and humidity (50 ± 10%). The animals had access to rat chow (type R36, Lantmännen, Kimstad, Sweden) *ad libitum* until the start of the rGT. During the rGT, the rats were food restricted to 85% of their free feeding weight and maintained on 14 g of rat chow given 1 h after their gambling session. The chow was spread out in the cage in order to secure access for both individuals in a pair. Body weight of the animals was closely monitored to ensure that the food restriction was properly carried out. Water was available *ad libitum* during the whole experiment. All procedures were performed during the dark phase of the light/dark cycle.

All animal experiments were approved by the Uppsala Animal Ethical Committee (permit number 5.8.18-00833/2017) and followed the guidelines of the Swedish Legislation on Animal Experimentation (Animal Welfare Act SFS 1998:56 and Animal Welfare Act SFS 2018:1192), and the European Union Directive on the Protection of Animals Used for Scientific Purposes (Directive 2010/63/EU).

#### Rat gambling task procedure

The rGT procedure has been described in detail elsewhere (Tjernström et al., 2022). The rGT took place in five-hole operant chambers (34 × 33 × 33 cm) placed inside ventilated sound-attenuating cabinets (56 × 56 × 70 cm; Med Associates, St. Albans, VT, United States). The chambers included response holes, a food tray, and a house light. Both the response holes as well as the food tray were equipped with stimulus lights and photograph beams to record responses. The food tray was connected to a pellet dispenser that delivered 45 mg sucrose pellets (Sandown Scientific, Middlesex, UK). The chambers were controlled by software written in Med PC (Med Associates, Inc.). The chambers were cleaned with 10% ethanol solution and allowed to dry between subjects.

The rats were habituated to the chambers on two daily 30-min sessions where sugar pellets were placed in all four response holes and in the food tray. Following this, the rGT training started and the rats had to progress through six levels of increasing complexity. This part of the training is intended to teach the rats to connect a response in the response hole with a pellet delivered in the food tray, and to increase the speed of which they perform the task. The training schedule was based on the schedule published by Zeeb et al. (2009), but with some modifications. A response in the food tray was needed to start a trial. When the training was completed a forced choice rGT was performed, that had all the same parameters as the free choice rGT (described in the following section). The difference was that in the forced choice rGT only one response hole was lit and only a response in that hole gave rise to either pellet deliveries or a timeout punishment. This was done for seven sessions to make sure that all the choice alternatives had been explored.

**FIGURE 1:**
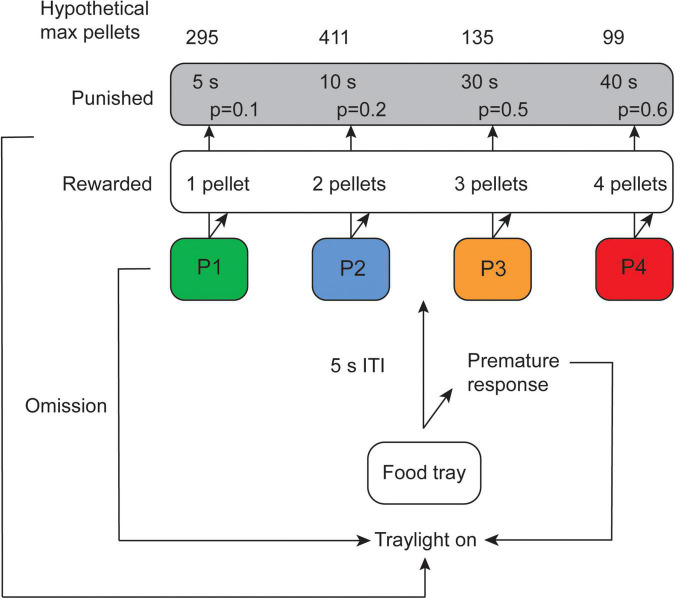
Schematic of the rat gambling task (rGT) displaying contingencies with regard to number of rewarded pellets as well as probability and duration of punishing timeouts for the different options (modified from Tjernström et al., 2022). Omissions and premature responses in the rGT have been described elsewhere (Tjernström et al., 2022; Tjernström and Roman, 2022; manuscript resubmitted for review) but were not used in the present study. ITI, inter-trial-interval.

A schematic of the test is shown in Figure 1. A trial was initiated by a response in the illuminated food tray, followed by a 5 s inter-trial-interval (ITI) when the subject had to wait before a response could be made. Thereafter, the rat was able to make a free choice between the four different holes. The response holes were associated with different number of pellets delivered, length of punishing timeouts and probabilities of reinforcer or punishment. The contingencies with regard to pellet probability, number of pellets delivered and duration of punishing timeouts for the different options were: P1: *p* = 0.9, 1, and 5 s; P2: *p* = 0.8, 2, and 10 s; P3: *p* = 0.5, 3, and 30 s; P4: *p* = 0.4, 4, and 40 s (Figure 1). The task was performed for five consecutive days per week and the sessions lasted for 30 min. The percentage of each choice was calculated [(number of choices of that option/number of completed trials) × 100] for P1, P2, P3, and P4.

### Data processing

In the IGT, a total of six participants were excluded from analysis due to repeated selections from one single deck across the entire session resulting in insufficient exploration of the deck contingencies assumed to guide decisions (Bechara et al., 1994), leaving a total of 264 human participants included in the analyses. In the rGT, two rats were excluded from end performance measures due to missing values in week 5.

### Statistical analysis

Statistical analyses were performed using IBM SPSS Statistics version 26 and Microsoft Excel. Figures were created in SPSS and GraphPad Prism 9. The significance threshold was set at *p* < 0.05. Normative scoring approaches were performed to analyze human and rat behavioral data. First, a standard scoring approach was conducted to assess overall performance in both tasks. Scores were calculated by the selections from the advantageous choices minus the selections from the disadvantageous choices [IGT: (C + D)–(A + B), and rGT: (P1 + P2%)–(P3 + P4%)] across 100 trials in both tests, and 25 days in the rGT. To investigate changes in patterns of decision-making during the tasks, choice scores were calculated for each 20-trial block across 100 trials in both tasks, and for each week (5 days) in the rGT. To standardize, block-wise scores in the IGT were multiplied by 5 to approximate the choice scores in rGT. Human and rat decision-making performance were also assessed according to their end performance. In the IGT, choice scores were obtained during the last 40 trials of the task (trials 61–100) which is commonly termed decision-making under risk (Brand et al., 2007). Correspondingly, choice scores in the rGT were obtained during the last week (5 days). Choice scores >0 indicated a tendency toward the advantageous choices, whereas choice scores <0 indicated a tendency toward the disadvantageous choices. Individual decision-making strategies during end performance were also explored by the formation of subgroups. The most extreme individuals in each choice were identified using mean + 1 standard deviation (SD) of the percentage of individual choices during trials 61–100 of the IGT, and during the last week (5 days) of the rGT. These were further categorized into subgroups of “good,” “intermediate,” and “poor” decision-makers according to the following: IGT: high in A + high in B = poor, high in C + high in D = good, intermediates = intermediates; rGT: high in P3 + high in P4 = poor, high in P1 + high in P2 = good, intermediates = intermediates. Statistical comparisons between the tasks were performed with regards to block-wise choice scores across the first 100 trials, and scores between subgroups during end performance. Repeated-measures analysis of variance (ANOVA) were used to analyze summary measures of choice scores, with significance threshold set at *p* < 0.05, and Huynh-Feldt and Greenhouse-Geisser corrections used. *Post-hoc* tests included Bonferroni corrections. The magnitude of the effect (η*_*p*_*^2^) in the ANOVA models were assessed according to Cohen (2013) guidelines: small = 0.01, medium = 0.06, and large = 0.15.

Analyses based on various personality characteristics within the human sample were performed using independent samples *t*-test. To explore whether participants decision-making could be explained by the loss-frequency decision-making strategy, scoring was obtained by taking the number of choices from the low-loss frequency decks (deck B + D) minus the high-loss frequency decks (deck A + C). The loss-frequency-based scoring approach was only explored in the human sample.

## Results

### Overall performance indicated by standard scoring approaches

#### Iowa gambling task (across 100 trials)

Choice scores were calculated according to the standard scoring approach assessing long term decision-making, taking the number of advantageous choices (deck C + D) minus the number of disadvantageous choices (deck A + B) across 100 trials. Using a cutoff of choice scores >0 most of the subjects (*N* = 147, 55.7%) showed a preference for the advantageous decks (C + D) across 100 trials, although a large proportion (*N* = 117, 44.3%) did not.

Overall performance was further investigated by calculating choice scores according to the loss-frequency approach. Using a cutoff of >0 a majority of the participants showed a preference for the low-loss frequency decks across 100 trials (*N* = 163, 61.7%), although a large proportion of the participants did not (*N* = 101, 38.3%) (Supplementary Table 1).

Independent sample *t*-tests were performed to investigate differences in choice scores across the task (trials 1–100) based on participants sex and self-reported symptoms on the ASRS-18, DSRS, AAS-15, and the PGSI. Results revealed no significant differences in performance related to sex or any of the self-reported symptoms. However, there was a numerical difference in mean choice scores between females and males. Individuals above the cutoff for self-rated symptoms of depression had lower mean choice scores, but the difference was not significant (Table 2).

**TABLE 2:** Independent sample *t*-test analysis of mean choice scores across the task (trials 1–100) based on sex and self-reported symptoms.

Sex	*N*	Mean choice score (trials 1–100)	*p*
Male	126	12.33	
Female	138	9.06	
			0.344
ASRS-18*			
ASRS self-rated symptoms <4	230	10.58	
ASRS self-rated symptoms ≥4	34	10.88	
			0.954
DSRS*			
DSRS self-rated symptoms <5	198	11.79	
DSRS self-rated symptoms ≥5	66	7.12	
			0.242
AAS-15*			
AAS self-rated symptoms <12	148	10.26	
AAS self-rated symptoms ≥12	116	11.09	
			0.812
PGSI*			
PGSI self-rated symptoms 0	230	10.11	
PGSI self-rated symptoms ≥1	34	14.06	
			0.444

*ASRS-18, Adult ADHD Self-Report Scale (18 item); DSRS, Depression Self-Rating Scale; AAS-15, The Adult Anxiety Scale (15 item); PGSI, Problem Gambling Severity Index. Significance threshold = *p* < 0.05.

#### Rat gambling task (across 25 days)

Choice scores [(P1 + P2%)–(P3 + P4%)] were calculated across all 25 days in the rGT. Using a cutoff of choice scores >0, all subjects (*N* = 72, 100%) showed a preference for the advantageous choices (P1 + P2) across all 25 days of the rGT.

### Performance patterns indicated by standard scoring approaches

#### Iowa gambling task (20-trial blocks)

To explore learning during the task, choice scores were calculated [number of advantageous choices (deck C + D) minus the number of disadvantageous choices (deck A + B)] for each 20-trial block (Table 3). The range of possible choice scores was −100 to +100 for each block. A repeated measures ANOVA with Huynh-Feldt correction determined a main effect of blocks on choice scores (*F*(3.160, 830.95) = 30.454, *p* < 0.001, η*_*p*_*^2^ = 0.10). *Post-hoc* test with Bonferroni corrections revealed a significant increase in choice scores from block 1 to block 3 (*p* < 0.001). There was no significant difference between block 3 and 4 (*p* = 0.387) or blocks 4 and 5 (*p* = 0.382), indicating an overall stabilization of performance during the final two blocks (trials 61–100).

**TABLE 3:** Mean choice score [(C + D%)–(A + B%)] and standard deviation (SD) per 20-trial block of the IGT.

Block	*N*	Choice scores
Block 1	264	−6.02 (34.72)
Block 2	264	5.00 (36.47)
Block 3	264	12.77 (40.91)
Block 4	264	18.18 (43.89)
Block 5	264	23.18 (48.66)

Sex differences in choice score progression was explored using a two-way repeated measures ANOVA with Huynh-Feldt corrections. Results revealed a significant main effect of block (*F*(3.187, 835.086) = 31.183, *p* < 0.001, η*_*p*_*^2^ = 0.106), but not for sex (*F*(1, 262) = 0.900, *p* = 0.334, η*_*p*_*^2^ = 0.003). *Post-hoc* tests with Bonferroni corrections showed no significant differences in choice scores between males and females in blocks 1, 2, 3, or 4. However males had higher choice scores in block 5 (*p* = 0.040).

Block-wise choice scores were also calculated according to the loss-frequency approach (Supplementary Table 2). A repeated measures ANOVA with Greenhouse-Geisser correction showed no significant effect of blocks on the loss-frequency scores (*F*(2.902, 763.27) = 1.230, *p* = 0.298, η*_*p*_*^2^ = 0.005).

#### Rat gambling task (20-trial blocks and weeks)

Average choice scores were calculated during the first 100 trials for each 20-trial block of the rGT. Since the rats performed different number of trials each day of testing, additional trials from day 2 were added for the rats that did not reach 100 trials during day 1. The range of possible choice scores was −100 to +100 for each block (Table 4). A repeated measures ANOVA with Greenhouse-Geisser correction determined a significant effect of blocks (*F*(4, 284.000) = 7.626, *p* < 0.001, η*_*p*_*^2^ = 0.09). *Post-hoc* tests with Bonferroni corrections revealed a significant increase in scores from block 1 to 2 (*p* < 0.001), but there were no increases in scores between blocks 2, 3, 4, and 5.

**TABLE 4:** Mean choice scores [(P1 + P2%)–(P3 + P4%)] and standard deviation (SD) per 20-trial block of the rGT.

Block	*N*	Choice scores	Week	*N*	Choice scores
Block 1	72	36.25 (28.21)	Week 1	72	61.05 (21.37)
Block 2	72	49.93 (31.58)	Week 2	71	74.23 (17.12)
Block 3	72	52.85 (33.91)	Week 3	71	75.74 (18.73)
Block 4	72	50.90 (33.65)	Week 4	70	71.84 (25.63)
Block 5	72	56.46 (30.11)	Week 5	70	69.81 (29.22)

Average choice scores were also calculated for each week that the rats underwent testing in the rGT (Table 4). Since percentage of choices were used to calculate the choice scores, the range of possible choice score was −100 to +100 for each week. A repeated measures ANOVA with Greenhouse-Geisser correction determined a significant effect of weeks (*F*(2.031, 140.167) = 12.690, *p* < 0.001, η*_*p*_*^2^ = 0.15). *Post-hoc* test with Bonferroni corrections revealed a significant increase in choice scores from week 1 to week 2 (*p* < 0.001). However choice scores were numerically higher in week 3 compared to week 5 but did not reach a significant difference (*p* = 0.069).

#### Iowa gambling task and rat gambling task (comparing 100 trials)

**FIGURE 2:**
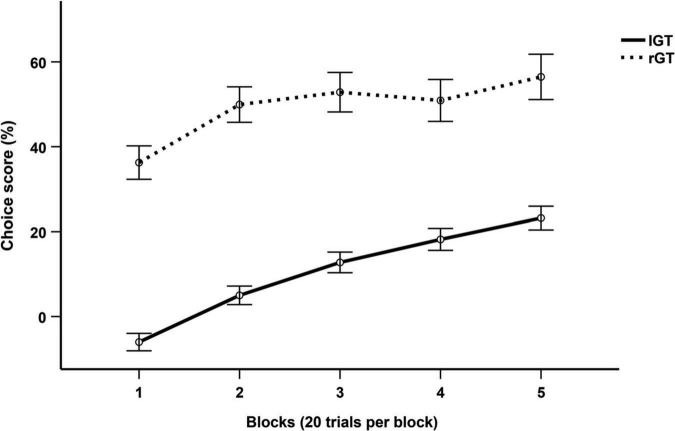
Mean choice score per 20-trial block across the first 100 trials of the IGT and rGT. Data are presented as mean ± 1 standard error (SE).

Differences in choice score progression between humans and rats were tested using a two-way repeated measures ANOVA with Huynh-Feldt corrections. There was a significant main effect of both block (*F*(3.283, 1096.389) = 19.571, *p* < 0.001, η*_*p*_*^2^ = 0.05) and group (*F*(1, 334) = 115.044, *p* < 0.001, η*_*p*_*^2^ = 0.256). *Post-hoc* tests with Bonferroni corrections determined a significant difference in choice scores between humans and rats for all blocks (*p* < 0.001) (Figure 2).

### Longitudinal choice patterns

#### Iowa gambling task (across 100 trials)

**FIGURE 3:**
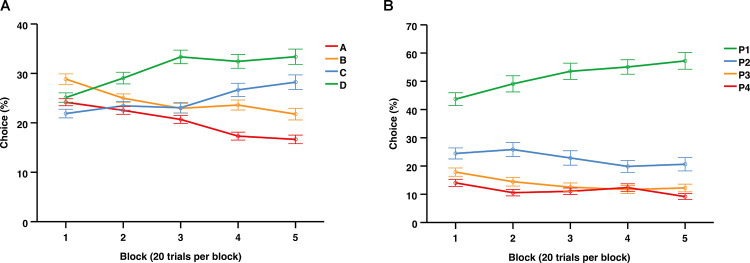
Average choices (%) per 20-trial blocks over the entire session of the IGT **(A)** and the first 100 trials of the rGT **(B)**. Data are presented as mean ± 1 standard error (SE).

Figure 3A illustrates changes in deck choices over time, indicating a trend toward the advantageous decks (C + D) away from the disadvantageous decks (A + B). During the final block of the IGT, the choice preferences (mean and SD) were the following: D = 32.90% (21.75), C = 27.44% (21.76), B = 22.68% (16.13), A = 16.98% (11.78).

Independent samples *t*-test were also conducted to explore sex differences in each separate choice option per block. Results revealed higher mean choices of B during the last block among females compared to males (*p* = 0.038). No differences were found in mean choices of A, C, or D across the blocks.

#### Rat gambling task (across 100 trials)

Figure 3B illustrates the average choices over time across the first 100 trials of the rGT, indicating an increasing preference for P1 and relatively stable choice levels for P2, P3, and P4. During the final block the choice preferences (mean and SD) were the following: P2 = 57.42% (21.38), P1 = 27.47% (15.55), P3 = 9.03% (11.14), P4 = 6.05% (11.49).

#### Rat gambling task (across 25 days)

**FIGURE 4:**
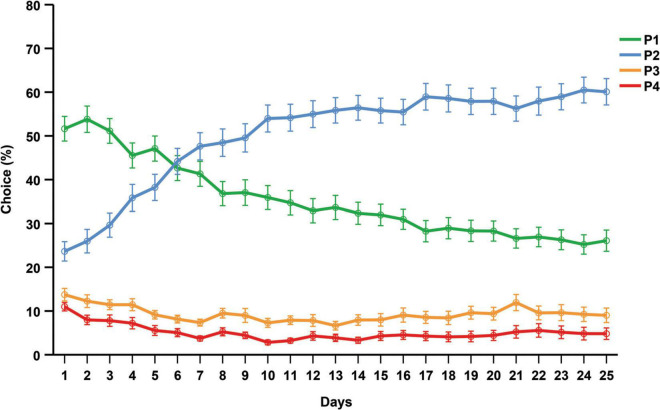
Average choices (%) for each day that the rGT was performed. Data are presented as mean ± 1 standard error (SE).

Figure 4 illustrates the average choices over the 25 days that the rGT was performed. During the first 6 days P1 was the favored choice, but from day 7 until the end of the rGT, P2 was the most preferred choice. During the last week of the rGT, the choice preferences (mean and SD) were as follows: P2 = 57.42% (21.38), P1 = 27.47% (15.55), P3 = 9.03% (11.14), P4 = 6.05% (11.49).

### Decision-making during end performance

#### Iowa gambling task (during trials 61–100)

Based on previous analysis (Section “Iowa gambling task (20-trial blocks)”) indicating stabilization in overall choice scores during the final two blocks, end performance was assessed by calculating choice scores across trials 61–100. Results revealed that most participants had developed a preference for the advantageous decks (*N* = 166, 62.9%) using a cutoff of >0. However, a noteworthy proportion of participants did not, thus failed to develop a preference for the long-term advantageous decks (*N* = 98, 37.1%).

End performance was also investigated by calculating loss-frequency scores. During this phase most participants showed a preference for the low-loss frequency decks, but results did not confirm a learned preference for the loss-frequency strategy in favor of the long-term strategy in our sample (Supplementary Tables 1, 2).

Independent sample *t*-tests were performed to investigate differences in standard choice scores during the last blocks (trials 61–100) based on participants’ sex and self-reported symptoms of ADHD, depression, anxiety, and problem gambling. Results revealed no significant differences in performance related to sex or any of the self-reported symptoms during this phase (Supplementary Table 3).

#### Rat gambling task (during the last 5 days)

Calculating choice scores [(P1 + P2%)–(P3 + P4%)] across the last 5 days of the task, i.e., the last week, revealed that most individuals had developed a preference for the advantageous choices (*N* = 66, 94.3%) using a cutoff of >0. The remaining four subjects preferred the disadvantageous choices (*N* = 4, 5.7%).

### Individual choices during end performance

#### Iowa gambling task (during trials 61–100)

**FIGURE 5:**
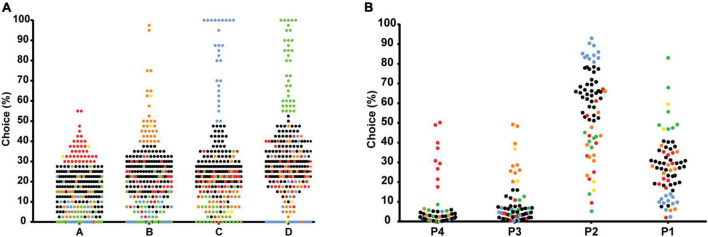
Scatterplots of the percentage of individual deck choices during trials 61-100 in the IGT **(A)**, and during week 5 of the rGT **(B)**, separating the most extreme individuals in each choice using mean ± 1 standard deviation. The most extreme individuals in each choice of the IGT **(A)** are colored as follows: high A-red, high B-orange, high C-blue, high D-green, high in both A and B-yellow, intermediates-black. The most extreme individuals in each choice of the rGT **(B)** are colored as follows: high P4-red, high P3-orange, high P2-blue, high P1-green, high in both P1 and P3-yellow, and intermediates-black.

Figure 5A illustrates the individual deck choices during the last part of the task (trials 61–100). Subgroups of individual choices were formed for each separate choice option during end performance. These subgroups were identified using the mean + 1 SD of the percentages in each separate choice option. This allowed identification and separation of the most extreme individuals in each choice, compared to the group mean. The proportion of individuals categorized as extremes in each choice option was A: *N* = 38 (14.4%), B: *N* = 31 (11.7%), C: *N* = 27 (10.2%), and D: *N* = 37 (14.0%). Individuals below the threshold of +1 SD in all choices were considered intermediates *N* = 129 (48.9%). Two individuals had a high frequency of choice in both A and B and were categorized accordingly. Subgroups were then plotted for visualization (Figure 5A). The mean percentages of individual deck choices for each subgroup of extreme individuals are displayed in Table 5.

**TABLE 5:** Mean percentages of individual deck choices (trials 61–100) in the IGT for each subgroup of extreme individuals in each choice based on +1 standard deviation (SD).

Group	*N*	A%	B%	C%	D%
A +1 SD	38	36.12	19.47	19.41	25.00
B +1 SD	31	11.53	52.34	14.52	21.61
C +1 SD	27	5.09	3.98	81.20	9.72
D +1 SD	37	6.96	8.45	8.65	75.95
A and B +1 SD	2	35	55	5	5
Intermediate <1 SD	129	17.73	23.99	27.40	30.87

#### Rat gambling task (during the last week)

The individual choices during week 5 (average of 5 days) are shown in Figure 5B. The proportion of individuals categorized as extremes in each choice option using mean + 1 SD were P1: *N* = 7 (10.0%), P2: *N* = 11 (15.7%), P3: *N* = 9 (12.9%), and P4: *N* = 9 (12.9%). Individuals below the threshold of +1 SD in all choices were considered intermediates *N* = 32 (45.7%). Two individuals had a high frequency of choice in both P1 and P3 and were categorized accordingly (Figure 5B). The mean percentages of individual deck choices for each subgroup of extreme individuals are displayed in Table 6.

**TABLE 6:** Mean percentages of individual deck choices (week 5) in the rGT for each subgroup of extreme individuals in each choice based on +1 standard deviation (SD).

Group	*N*	P1%	P2%	P3%	P4%
P1 +1 SD	7	57.01	32.54	6.72	3.74
P2 +1 SD	11	9.79	85.63	3.41	1.17
P3 +1 SD	9	24.41	41.33	32.27	1.98
P4 +1 SD	9	23.76	39.41	3.25	33.59
P1 and P3 +1 SD	2	53.21	17.98	28.63	0.18
Intermediate <1 SD	32	27.38	65.22	5.32	2.00

#### Sub-group comparisons during end performance

**FIGURE 6:**
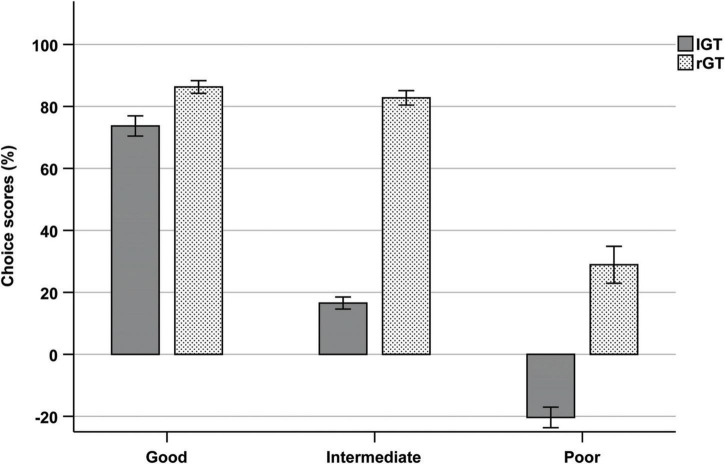
Mean choice scores of each subgroup (good, intermediate, and poor) during trials 61–100 of the IGT and week 5 of the rGT.

To enable comparisons between the two tasks, extreme individuals in each choice option were categorized into subgroups of “good,” “intermediate,” and “poor” decision-makers. IGT: high in A + high in B = poor, high in C + high in D = good, intermediates = intermediates; rGT: high in P3 + high in P4 = poor, high in P1 + high in P2 = good, intermediates = intermediates. The two individuals categorized as high in both A and B in the IGT were considered “poor.” The two individuals categorized as high in P1 and P3 in the rGT were considered “intermediates.” The proportion of individuals in each subgroup of the IGT was as follows: good: *N* = 64 (24.2%), intermediate: *N* = 129 (48.9%), and poor: *N* = 71 (26.9%). The proportion of individuals in each subgroup of the rGT was as follows: good: *N* = 18 (25.7%), intermediate: *N* = 34 (48.6%), poor: *N* = 18 (25.7%). A one-way ANOVA was used to compare choice scores between groups during end performance. Results showed a significant main effect of group (*F*(5) = 179.846, *p* < 0.001, η^2^ = 0.733). *Post-hoc* tests with Bonferroni corrections showed no differences in choice scores between the subgroup of good decision-makers in the IGT and good decision-makers in the rGT (*p* = 0.872), as well as intermediates in the rGT (*p* = 1.000). Intermediates in the IGT had significantly lower choice scores than intermediates in the rGT (*p* < 0.001). Poor decision-makers in the IGT also had significantly lower choice scores that the poor decision-makers in the rGT (*p* < 0.001) (Figure 6).

## Discussion

This study explored decision-making strategies among humans in the IGT and rats in the rGT, using similar approaches to compare both group and individual behavioral data, highlighting differences and similarities in the two tasks. Both the IGT and rGT results are based on large sample sizes. Differences were evident on a group level, in terms of overall performance and changes in average choice preferences across the tasks. On average, rats showed relatively consistent pronounced preferences for the advantageous choices throughout the entire task, while human performance was characterized by both exploration and learning as the task progressed. This confirms the typical performances of humans and rats previously reported (Bechara et al., 1994; Zeeb et al., 2009), and highlights the effect of procedural differences between the tasks. Nevertheless, the results also indicated similarities in terms of subgroup variability in choice preferences during end performance in both species.

### Overall performance, choice progression and end performance

Most human participants increasingly preferred the advantageous choices, consistent with the assumption that most healthy participants learned to choose the advantageous options (Bechara et al., 1994, 2001; Bechara and Damasio, 2002). A distinct pattern of preference for the two advantageous options over the two disadvantageous options started to emerge and stabilize during the final part of the task, consistent with current theories that more conscious and deliberate choices are made during this phase, i.e., decisions under risk (Maia and McClelland, 2004; Dunn et al., 2006; Brand et al., 2007; Guillaume et al., 2009). Nevertheless, a substantial proportion of participants showed an overall preference for the disadvantageous options across the task and during end performance. Ultimately, the average number of advantageous selections in the current study did not reach the same high levels as originally reported by Bechara et al. (1994), but were within the same range (50–60%) as usually reported for healthy individuals in more recent studies (Steingroever et al., 2013; Buelow and Blaine, 2015). This is consistent with the notion that the original net-scores by Bechara et al. (1994) rarely have been replicated in healthy samples [for a review, see Steingroever et al. (2013)].

In contrast, all rats in the rGT showed a pronounced overall preference for the advantageous choices across all sessions, consistent with studies showing that a majority of rats learn and maintain a stable choice of P2 (Zeeb et al., 2009; Zeeb and Winstanley, 2011; Barrus et al., 2015; Barrus and Winstanley, 2016; Tjernström et al., 2022; Tjernström and Roman, 2022; manuscript resubmitted for review). The choice progression over time in the rGT showed a markedly different pattern relative to the progression in the IGT. The only shift in choice preferences among the rats was from P1 to P2 after day 7. Average choices of P3 and P4 was consistent throughout the 25 days, leading to a choice score that was almost unchanged throughout the course of the experiment. Consequently, only a small proportion of the rats were classified as disadvantageous during the final week of testing. Most rGT studies report the choice preference or the average score at the end of the rGT when the choice behavior is stable. Reported choice scores for rats in the rGT vary considerably; some report that disadvantageous individuals make up around 20% of the population (Adams et al., 2017b; Ferland and Winstanley, 2017), others over 30% (Ferland et al., 2018; Tremblay et al., 2021) or 0% (Ferland et al., 2019). However, reports on the weekly progression of choices are scarce and early studies report inconsistencies regarding the level of choice progression between P1, P3, and P4 (Zeeb et al., 2009; Zeeb and Winstanley, 2011). Consequently, previous research also revealed inconsistencies in terms of the ranking of these choices during end performance among rats (Baarendse et al., 2013; Barrus et al., 2015; Barrus and Winstanley, 2016). Therefore, it is difficult to discern how choice patterns normally develops and to what extent they differ between populations. The initial preference for P1 in the present study might be caused by the initial part of the training protocol, prior to the forced choice training. During this part a correct response yields one sugar pellet (the same as for choosing P1). Hence, the rats may initially choose P1 since it is similar to the conditions they are used to, or they might be avoiding timeouts at any cost before understanding that P2 is the more advantageous option. The initial preference for P1 agrees with previous findings (Tjernström et al., 2022; Tjernström and Roman, 2022; manuscript resubmitted for review), but contrasts findings of others (Zeeb et al., 2009; Zeeb and Winstanley, 2011). The inconsistent results on choice preference, as well as the differences in development of choice patterns, highlight the need for baseline data on choice progression to be reported. This may generate important insights regarding the behavioral effects of strain differences or small differences in training protocol, as well as underlying neurobiological mechanisms.

The differences in choice progression between humans in the IGT and rats in the rGT may be attributed to four procedural differences: (1) effects of rGT pre-training, (2) the number of trials administered during the two tasks, (3) the probabilistic schedule of the tasks, and (4) the win/loss frequency and magnitude. In the IGT, the present results reveal that trial-and-error learning occurred during the first 60 trials, consistent with the assumption that participants explore the different options during the first part of the task (decisions under ambiguity) and progressively develops a stable preference during the later phase of the task (decisions under risk). In the rGT, the rats undergo training in the operant chambers prior to the rGT. The first, most time-consuming part of the training only teaches them to make responses and collect pellets and gives no information about the choices and their respective pellet/punishment contingencies. However, the subsequent seven-session forced choice training requires the rats to choose all four options to make sure that all options have been explored with regard to number of pellets delivered and duration of punishing timeouts. As pointed out by Zeeb et al. (2009) the choice preferences in the rGT are established early on due to the forced choice training and stabilizes with multiple testing. Hence, the forced choice training in the rGT relative to the verbal instructions given to the humans in the IGT, may have an impact on choice progression in the two tasks.

Additionally, humans performed 100 trials in one session while the rats performed 25 sessions. The rGT procedure, with testing across multiple sessions, enable the rats to reach stability in their choice preferences. Hence, humans and rats likely rely on different memory systems to guide their choices. While humans must rely on working memory during one session, the rats likely use their long-term memory to determine their strategy after repeated testing. Consequently, different neural systems might be active during task performance in humans and rats. Several researchers have shown that a large proportion of healthy participants in the IGT fail to progress from the initial exploration phase and develop a stable preference after 100 trials, but that many were able to achieve a better final performance when the number of trials were increased to 200 trials (Balodis et al., 2006; Buelow et al., 2013; Steingroever et al., 2013; Bull et al., 2015; Cabeza et al., 2020). Therefore, an important limitation is that the task cannot dissociate learning insensitivities from risky preferences. Risks and benefits of each choice becomes explicit for some individuals toward the end of the task, but for others trial-by-trial learning still occurs after 100 trials. Hence, the IGT provides information on individual differences in decision-making during uncertain conditions, but the ability for IGT to distinguish pronounced risk-taking profiles may be questioned. Increasing the number of trials in the IGT would therefore enable analysis of individual differences in both trial-and-error learning as well as a more stable final performance reflecting individual choice preference, like the rGT.

Furthermore, the IGT and rGT also differ in terms of their probabilistic schedules. In IGT, the decks are stacked in a fixed order, and the large losses associated with the disadvantageous options occur after several trials (especially in deck B). This may prompt an initial preference for the disadvantageous choices that switches to a learned preference for the advantageous choices throughout the session. However, in the rGT the losses occur randomly. Previous research found that changing the order of the decks in the IGT, so that the large losses of the disadvantageous decks are presented on earlier trials, lead participants to favor the advantageous decks from the first block (trials 1–20) (Fellows and Farah, 2005). The effects of the rats forced choice training and the differences in the rGT win/loss contingency schedules likely contribute to the way the choices are perceived and implies that the degree to which rats and humans base their choices on explicit knowledge or implicit guidance during these tasks may differ significantly. The extent to which the individuals in the rGT and IGT, respectively, are explicitly aware of the basis of their decisions, is highly relevant for the interpretation of the processes involved in the tasks.

The rGT is often presented as the rat analog of the IGT, but the win/loss frequency and magnitude associated with each option differ between the tasks. It is mainly P1 in the rGT that differs from the options available in the IGT. Choosing P1 will give rise to frequent small wins and infrequent small losses, which may reflect risk averse decision-making (de Visser et al., 2011). It is unclear whether this type of risk aversive decision-making component is represented in the IGT. Furthermore, the most preferred advantageous choices in the rGT (P2), and in the IGT (deck D), may not reflect similar motives among rats and humans. Repeated selections from P2 may indicate a reward maximization strategy. However, the motives underlying repeated selections from deck D (containing relatively small infrequent losses) is not as obvious and may reflect either reward maximization or loss aversion.

However, previous research has shown that some individuals in the IGT may apply a decision-making strategy where they seek to minimize the frequency of losses, rather than maximizing the long-term gains of the different choices. Several studies report this strategy among healthy individuals (Caroselli et al., 2006; Lin et al., 2007; Chiu et al., 2008; Steingroever et al., 2013; Barnhart and Buelow, 2021). The loss-frequency scoring approach was explored in the human sample, but results did not confirm a learned preference for the low-loss-frequency strategy in favor of the standard long-term strategy in our sample.

Given that the magnitude and frequency of the choices in the IGT and rGT differ, they do not entirely correspond in terms of their risk/gain potential in the short- and long-term. Hence, there may be a discrepancy in terms of the way they are perceived and processed. To our knowledge, this has not been addressed in previous research, but is relevant from a translational perspective since it begs the question of what defines individuals as good or poor decision-makers in each of the tasks. Another version of the rGT has been developed to specifically measure IGT-like decision-making during only one session, in order to track individual choices and ongoing decision-making processes, that more closely resemble the IGT paradigm (Rivalan et al., 2009). Hence, comparing IGT-like decision-making processes in humans and rats utilizing this version of the rGT may represent an important future direction for research on decision-making. There is also a cued version of the rGT available, in which win-related audiovisual cues are incorporated. These cues are added to more closely mimic the environment present in human gambling and were shown to increase risky choice in the rGT (Barrus and Winstanley, 2016). The use of this version might be suitable when the aim is to specifically investigate gambling-like behavior, and it would be interesting to compare the individual differences in choice behavior in rGT and the cued rGT, as well as the IGT.

### Individual choices during end performance

The individual choices during end-performance were also examined in both the IGT and rGT, by the formation of subgroups including the most extreme individuals in each choice. Despite differences in terms of the overall level and distribution of choices, there were also similarities in terms of subgroup variability in choice preferences. In both tests, extreme individuals were found in each of the different options and the two advantageous options did not seem to correspond with each other, i.e., individuals preferred either one of the advantageous options, but not both. The choice score comparisons of good, intermediate and poor decision-makers also revealed similar level of performance among the good decision-makers in IGT and rGT. However, there was a slightly different pattern for extreme individuals of the two disadvantageous options between the tasks. For individuals in the IGT with the highest choice frequencies of A and B, choices were somewhat evenly distributed between the other options, while extreme individuals in P3 and P4 still had an overall preference for P2. Hence, poor decision-makers in the rGT performed considerably better than poor decision-makers in the IGT. In addition, the level of performance of intermediates were also higher in the rGT than IGT due to the contrasting levels of developed preferences by the end of the tasks. These results once again highlight differences in the level of performance between the tasks, but also interesting variations in combinations of choice preferences beyond choice score performances. This type of subgroup variability, including the relationships between the different choices, is not generally displayed, or discussed in either IGT or rGT studies, so it is unclear whether this is a universal finding or if other groups would find a different relationship between the options. Furthermore, aggregating advantageous and disadvantageous choices only allows two groups of “advantageous” or “disadvantageous” individuals based on positive or negative scores and fails to identify variations in choice behavior, as well as underlying neurobiological mechanisms. In both tasks, the groups that are formed with the standard scoring approaches evidently consist of individuals with highly contrasting choice patterns. In the rGT, the overall low preference for any of the disadvantageous options results in very few individuals that are classified as disadvantageous. However, when looking at the individual choice data there are several individuals with a much higher preference for these options compared to the rest of the population. Hence, analysis of individual choice preferences in favor of the choice score approach, may reveal widely different strategies guided by various underlying decision-making processes.

Some rGT studies discuss individual differences in choice behavior without aggregating advantageous and disadvantageous choices. One study found that animals with high and low motor impulsivity differed in their preference of the different choices; animals in the low motor impulsivity group chose P2 more, animals in the high motor impulsivity group chose P1 and P4 more, while choice of P3 did not differ (Barrus et al., 2015). This relationship between choice preferences corroborates our finding that options P1 and P2, that in standard scoring approaches are combined, does not seem to represent the same thing for the animals (Vonder Haar et al., 2022). A recent study used cluster analysis to find choice phenotypes and found five distinct clusters: one with strong P2 preference, one with moderate P2 preference, one P3-preferring, one P4-preferring, and lastly a group with P1 and P3 preference (Vonder Haar et al., 2022). Once again this indicates that the relationship between the choices in the rGT is not as simple as P1 and P2 being perceived as advantageous and P3 and P4 as disadvantageous.

Another factor that may contribute to why the rats are able to clearly distinguish between the advantageous and the disadvantageous choices early on concerns the modeling of “wins” and “losses” which remains a major challenge in animal models. Money is a secondary reinforcer, and its incentive value is highly subjective. However, food, and especially palatable food, is a primary reinforcer influenced by factors such as hunger and satiety, as well as rewarding properties (de Visser et al., 2011). Cabeza et al. (2020) argued that the faster learning rate among mice compared to humans observed in their study might, in part, be explained by the nature of reinforcers affecting the basic level of motivational states in rodents and humans. Secondly, although both tasks assess decision-making strategies of overall maximization, the signaling of losses in the two tasks differ. Ultimately, rats and humans base their strategies on different types of punishment feedback. It is possible that the reinforcing/punishing incentives of “wins” and “losses” in the two tasks adds to the difference in performance between the humans and rats of this study, in addition to procedural differences concerning the pre-training forced choice and the probabilistic schedules of the tasks.

This study compared a population-based sample of humans with outbred male Lister Hooded rats and complement a previous comparative study in which inbred mice and a different version of gambling task were used (Cabeza et al., 2020). The rat sample used herein is genetically diverse and is therefore expected to exhibit large individual differences, but the individual variability in the human sample is presumably larger also due to different environmental factors. Humans in the current study were not controlled for possible contributors known to affect IGT performance, such as diagnosed psychiatric conditions or neuropsychiatric disabilities (Buelow and Suhr, 2009). However, analyses were performed to investigate differences in overall and end performance based on self-reported symptoms of ADHD, depression, and anxiety, where no significant differences were found. In agreement, a thorough behavioral characterization found no specific behaviors of relevance to exploration, risk assessment, risk taking and shelter seeking to be associated with later gambling strategies in rats (Tjernström et al., 2022). Furthermore, while the rat sample comprised males only, the human sample included both males and females in order to maintain sufficient power. Previous research found that females generally perform worse than males in the IGT, possibly due to increased sensitivity to the frequency of losses among females. As a consequence, females generally require additional trials before they reach similar levels of performance as males (van den Bos et al., 2013). In the current study males and females’ performance differed during the last block due to a higher preference for deck B among females compared to males. There was also a numerical difference in mean choice scores between males and females, but this did not reach significance. Studies comparing decision making processes in male and female rats are scarce (Orsini and Setlow, 2017), and therefore represent an important addition to pre-clinical studies of decision-making. Using a similar rGT as herein it was found that females developed an optimal choice behavior more rapidly than males (Peak et al., 2015), while a study in which a different gambling task was used found the opposite result (van den Bos et al., 2012). Furthermore, performance of the human sample may also relate to the age of the study participants. Developmental studies consistently report age-related improvements in advantageous decision-making on the IGT (Crone and van der Molen, 2004; Hooper et al., 2004; Cassotti et al., 2011; Beitz et al., 2014). Although, there is no clear consensus regarding the age span of adolescence, individuals above the age of 20 are usually defined as adults [for a review, see Defoe et al. (2015)]. The human sample herein, comprising young adults between the age of 18–21, are in the midst of transition into early adulthood, and may have varying degrees of cortical and sub-cortical maturation and activity affecting decision-making (Hooper et al., 2004), as well as responses to reinforcers (Braams et al., 2015).

## Conclusion

The overall results confirmed the typical decision-making patterns usually reported separately in both humans and rats. Most healthy humans and rats learn to favor the advantageous choices associated with small losses and larger long-term gains, in favor of the immediate large reinforcers. However, to what extent humans and rats reach this level of performance during the tasks differ considerably. Consequently, the procedural differences between the tasks makes them suitable to study different aspects of decision-making. The IGT procedure can be used to track ongoing decision-making processes from exploration to exploitation as the task progresses, while the rGT with repeated daily sessions allows the identification of stable choice preferences in decision-making.

Despite the procedural differences between the two tasks, both the IGT and rGT revealed individual variability in choice preferences during end performance, as both humans and rats formed preferences for a single option or a combination of options. Consequently, the formation of different combinations of choice preferences may reflect widely different underlying mechanisms that drive decision-making on an individual level. In order to make inferences regarding the underlying mechanisms operating during these tasks, both human clinical, and pre-clinical research would benefit from more detailed analyses on individual variations in decision-making. Moreover, future research is needed to address the correlations between the separate choices in IGT and rGT, and whether they tap into similar processes (reward maximization or loss aversion) during reinforcement-based decision-making in humans and rats. This is a first attempt to increase the understanding of similarities and differences in the IGT and rGT and their respective strengths and limitations from an explorative perspective on decision-making processes, but further studies are needed.

## Data availability statement

The raw data supporting the conclusions of this article will be made available by the authors upon request.

## Ethics statement

The studies involving human participants were reviewed and approved by Ethical Review Board of Uppsala. The patients/participants provided their written informed consent to participate in this study. The animal study was reviewed and approved by Uppsala Animal Ethical Committee.

## Author contributions

CH and NT: investigation, data collection, formal analysis, visualization, write the first draft of the manuscript, and review and editing. SV: investigation, data collection, supervision, conceptualization, methodology, and review and editing. MR: investigation, formal analysis, visualization, and review and editing. KN: funding acquisition, supervision, and review and editing. ER and CÅ: funding acquisition, supervision, conceptualization, methodology, and review and editing. All authors contributed to manuscript revision, read, and approved the submitted version.
