# Supplementary material for: Exploring decision-making strategies in the Iowa gambling task and rat gambling task
Source: Front Behav Neurosci. 2022 Nov 3;16:964348. doi: 10.3389/fnbeh.2022.964348 (PMC9669572; doi:10.3389/fnbeh.2022.964348)
Supplement: Supplementary file 1 [file Data_Sheet_1.PDF]

Supplementary Material

1 Overall and end performance indicated by loss-frequency scores in the IGT

To explore whether decision-making in the IGT could be explained by the loss-frequency decision-making strategy, scoring was obtained by taking the number of choices from the low-loss frequency decks (B + D) minus the high-loss frequency decks (A + C). Using a cutoff of > 0 a majority of the participants showed a preference for the low-loss frequency decks across 100 trials (N = 163, 61.7 %), although a large proportion of the participants did not (N = 101, 38.3 %). End performance was also indicated by loss-frequency scores during trials 61–100. During this phase most participants showed a preference for the low-loss frequency decks (N = 158, 59.8 %), using a cutoff of > 0. Nevertheless, a noteworthy proportion of the participants still showed a tendency towards the high-loss frequency decks (N = 106, 40.2 %) during the last part of the task.

Table S1. Number and percentage of individuals in the high-loss/low-loss categories based on scores (cutoff > 0) across the entire task and end performance.

**Performance trial 1–100**	**N**	**%**	**Performance trial 61–100**	**N**	**%**
High-loss (scores < 0)	101	38.3	High-loss (scores < 0)	106	40.2
Low-loss (scores > 0)	163	61.7	Low-loss (scores > 0)	158	59.8
Total	264	100	Total	264	100

2 Loss-frequency scores per 20-trial block in the IGT

Block-wise scores in the were multiplied by 5 to approximate the choice scores in rGT, resulting in a range of possible choice scores between -100 to +100 for each block. During the initial part of the task (trials 1–40), participants showed higher scores in the frequency-based scoring than the standard long-term scoring, which indicates an initial tendency towards the low-loss frequency decks. However, during the subsequent trials, the frequency-based scores started to decline, while standard long-term scores increased, suggesting that the participants learned to prefer the long-term advantageous decision-making strategy.

Table S2. Mean loss-frequency score and standard deviation (SD) per 20-trial block of the IGT.

**Block**	**Loss-frequency scores [(B + D) - (A + C)]**
Block 1	7.90 (32.70)
Block 2	8.05 (34.30)
Block 3	12.55 (38.35)
Block 4	12.05 (43.50)
Block 5	10.25 (48.05)
Total score	50.85 (140)

3 Differences in choice scores during end performance based on sex and self-reported symptoms

Independent sample t-tests were performed to investigate differences in standard choice scores during the last blocks (trials 61–100) based on participants sex and self-reported symptoms on the Adult ADHD Self-Report Scale (ASRS) (Kessler et al., 2005), Depression Self-Rating Scale (DSRS) (Svanborg and Ekselius, 2003), The Adult Anxiety Scale-15 (AAS-15) (Spence, 2017), and the Problem Gambling Severity Index (PGSI) (Ferris and Wynne, 2001). Choice scores were multiplied by 2.5 to approximate the choice scores in rGT and overall scores (trials 1–100) in the IGT, resulting in a range of possible choice scores between -100 to +100. Results revealed no significant differences in performance related to sex or any of the self-reported symptoms, during this phase. Although, individuals above the cutoff for self-rated symptoms of depression, anxiety and ADHD had lower mean choice scores, the differences were not significant. No significant differences were found for self-reported symptoms of problem gambling. There was also a numerical difference in mean choice scores between females and males, but this difference did not reach significance.

Table S3. Independent sample t-test analysis of mean choice scores during end performance (trials 61–100) based on sex and self-reported symptoms.

**Sex**	**N**	**Mean choice score (%) (trial 61–100)**	***p***
Male	126	25.73	
Female	138	16.08	
			0.066
**ASRS (18 item)**			
ASRS self-rated symptoms < 4	230	21.25	
ASRS self-rated symptoms ≥ 4	34	16.9	
			0.576
**DSRS**			
DSRS self-rated symptoms < 5	198	23.53	
DSRS self-rated symptoms ≥ 5	66	12.13	
			0.056
**AAS (15 item)**			
AAS self-rated symptoms < 12	148	23.85	
AAS self-rated symptoms ≥ 12	116	16.65	
			0.167
**PGSI**			
PGSI self-rated symptoms 0	230	19.90	
PGSI self-rated symptoms ≥ 1	34	26.03	
			0.428

**ASRS-18* = Adult ADHD Self-Report Scale (18 item). *DSRS* = Depression Self-Rating Scale. *AAS-15* = The Adult Anxiety Scale (15 item). *PGSI* = Problem Gambling Severity Index.
Significance threshold = $p < 0.05$.
